# Effects of saxagliptin on early microvascular changes in patients with type 2 diabetes

**DOI:** 10.1186/1475-2840-13-19

**Published:** 2014-01-14

**Authors:** Christian Ott, Ulrike Raff, Stephanie Schmidt, Iris Kistner, Stefanie Friedrich, Peter Bramlage, Joanna M Harazny, Roland E Schmieder

**Affiliations:** 1 Department of Nephrology and Hypertension, University of Erlangen-Nuremberg, Ulmenweg 18, Erlangen, Germany; 2 Institut für Pharmakologie und präventive Medizin, Mahlow, Germany; 3 Department of Pathophysiology, Warmia Masury University, Olsztyn, Poland

**Keywords:** Saxagliptin, DPP-4 inhibitor, Type-2 diabetes, Retinal blood flow, Central hemodynamics

## Abstract

**Background:**

Patients with diabetes mellitus are at increased risk for microvascular complications. Early changes in microcirculation are characterized by hyperperfusion (e.g. in the retina and kidney) and increased pulse wave reflection leading to increased aortic pressure. We investigated the effects of the DPP-4-inhibitor saxagliptin on early retinal microvascular changes.

**Methods:**

In this double-blind, controlled, cross-over trial 50 patients (without clinical signs of microvascular alterations) with type-2 diabetes (mean duration of 4 years) were randomized to receive placebo or 5 mg saxagliptin for 6 weeks. Retinal arteriolar structure and retinal capillary flow (RCF) at baseline and during flicker-light exposure was assessed by scanning laser Doppler flowmetry. Central hemodynamics were assessed by pulse wave analysis.

**Results:**

Postprandial blood glucose (9.27 ± 0.4 versus 10.1 ± 0.4 mmol/L; p = 0.001) and HbA1c (6.84 ± 0.15 (51 ± 1.6) versus 7.10 ± 0.17% (54 ± 1.9 mmol/mol); p < 0.001) were significantly reduced with saxagliptin treatment compared to placebo. RCF was significantly reduced after treatment with saxagliptin (288 ± 13.2 versus 314 ± 14.1 AU; p = 0.033). This was most pronounced in a subgroup of patients (n = 32) with a fall in postprandial blood glucose (280 ± 12.1 versus 314 ± 16.6 AU; p = 0.011). No significant changes in RCF were seen during flicker-light exposure between placebo and saxagliptin, but the vasodilatory capacity increased two-fold with saxagliptin treatment. Central augmentation pressure tended to be lower after treatment with saxagliptin (p = 0.094), and central systolic blood pressure was significantly reduced (119 ± 2.3 versus 124 ± 2.3 mmHg; p = 0.038).

**Conclusions:**

Our data suggest that treatment with saxagliptin for 6 weeks normalizes retinal capillary flow and improves central hemodynamics in type-2 diabetes.

**Trial registration:**

The study was registered at (ID: NCT01319357).

## Introduction

Diabetes mellitus is associated with microvascular complications such as diabetic retinopathy and nephropathy [1,2]. Early vascular and hemodynamic changes, occurring prior to any clinical manifestation, are hyperperfusion of the retinal and renal circulation, vascular remodeling and an increase in pulse wave reflection leading to an increased aortic pressure [3-5]. The prevention of early microvascular changes due to glucotoxicity is a desirable goal in the treatment of diabetes mellitus.

Examination of the retinal circulation offers the unique opportunity to directly visualize and investigate the microvasculature in vivo non-invasively [6-9]. Scanning laser Doppler Flowmetry (SLDF) recently emerged as a reliable [10] and valid clinical tool [11] for early detection of these microvascular changes namely retinal hyperperfusion and early vascular remodeling of small retinal arterioles. The method is well established in clinical studies analyzing early vascular remodeling and hemodynamic changes due to hypertension [12-14].

Saxagliptin is a potent, selective, reversible, and competitive dipeptidyl peptidase-4 (DPP-4) inhibitor [15,16]. Saxagliptin increases the level of the incretin hormones glucagon-like-peptide 1 (GLP-1) and the glucose-dependent insulinotropic polypeptide (GIP). GLP-1 stimulates glucose-dependent insulin secretion and blocks the secretion of glucagon thus reducing fasting as well as postprandial glucose levels [17]. Infusion of GLP-1 has been reported to ameliorate endothelial dysfunction in patients suffering from coronary artery disease [18] and it was recently shown that infusion of GLP-1 into healthy human subjects increases both normal and acetylcholine-induced vasodilatation [19]. In studies on rats with diabetes, GLP-1 infusion nearly re-established their normal vascular tone [20] and there are further data from experimental animals that indicate a beneficial effect of GLP-1 on endothelial function [21]. In vitro demonstrated that DPP-4 is expressed in endothelial cells and the inhibition of DPP-4 reduced the microvascular tone through direct mediation of the nitric oxide (NO) system [22].

The aim of the **E**ffects of **S**axagliptin on **EN**dothelial function in patients with type-2 **Di**abetes (ESENDI)-study was to analyze the impact of saxagliptin on early microvascular changes due to type-2 diabetes by non-invasively measuring the retinal circulation, documenting hemodynamic changes and assessing early vascular remodelling.

## Methods

### Study design

ESENDI was a randomized, double-blind, placebo-controlled investigator sponsored cross-over trial conducted in Erlangen-Nuremberg, Germany between November 2010 and July 2012. The study protocol was approved by the Ethic Committee of the University of Erlangen-Nuremberg and the study was performed according to Declaration of Helsinki and “good clinical practice” (GCP) guidelines. Written informed consent was obtained from all patients before study entry.

The study was registered at clinicaltrials.gov, ID: NCT01319357.

### Study population

Patients of either gender and age between 18 and 75 years were eligible for inclusion into the study given they were diagnosed with type-2 diabetes mellitus (defined by fasting glucose ≥ 7.0 mmol/L or HbA1c ≥ 6.5% (48 mmol/mol) or receiving anti-diabetic pharmacotherapy). Selected exclusion criteria included being on more than one blood glucose lowering medication, insulin or (current or within the previous 6 months) treatment with any incretin-based treatment strategy such as DPP-4 inhibitors or GLP-1 agonists. Furthermore patients with micro- or macrovascular complications such as diabetic retinopathy, macroalbuminuria, an acute cardiovascular event (e.g. myocardial infarction), unstable angina or stroke within 6 months prior to enrollment were excluded. Female subjects of child bearing potential or within two years of the menopause were excluded unless a pregnancy test at the screening visit was negative and adequate contraceptive precautions made during the study.

### Objectives

The principal objective was to investigate the effect of saxagliptin compared to placebo on early vascular remodeling and on the retinal capillary flow (RCF). By applying SLDF, objectives of the study were therefore: to analyze RCF at baseline, after flicker light, and after i.v. NG-monomethyl-L-arginine (L-NMMA) application, as well as to assess wall to lumen ratio (WLR) of retinal arterioles 6 weeks after saxagliptin treatment compared to placebo. In addition we evaluated the effect of saxagliptin on carotid-to-femoral pulse wave velocity (PWV) and on central systolic pressure by aortic pulse wave contour analysis, and on urinary albumine-to-creatinine ratio (UACR). The effect of saxagliptin on metabolic parameters (HbA1c, glucose levels, adiponectin, lipids, insulin and HOMA index), was also measured.

It was pre-specified that results were to be validated in a subgroup of patients with a reduction of postprandial blood glucose, since the reduction of postprandial blood glucose is thought to represent a direct measure of the pharmacologic action of saxagliptin in humans.

### Treatment/intervention

**Figure 1:**
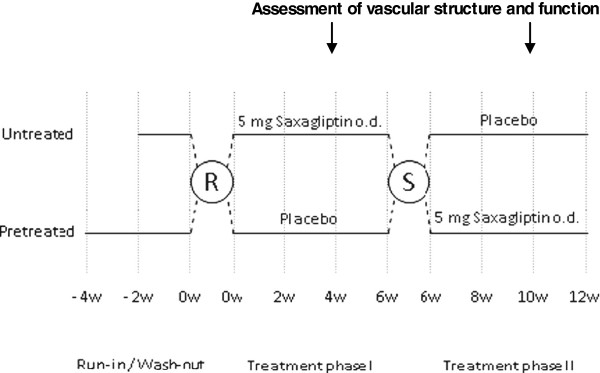
Study design.

All patients entered a run-in / wash-out phase of 4 weeks given they were on any prior anti-diabetic treatment and of 2 weeks if they were treatment-naïve (Figure 1). Patients were then randomly assigned to either 5 mg of saxagliptin once daily or matching placebo. At 6 weeks patient’s treatment was switched (cross-over) and treatment continued for another 6 weeks without a washout between treatment phases. The total treatment duration was 12 weeks.

### Measurement of retinal capillary flow and retinal arteriolar structure

RCF was assessed using SLDF at 670 nm (Heidelberg Retina Flowmeter, Heidelberg Engineering, Germany). Measurements were performed in the juxtapapillary area of the right eye, 2-3 mm lateral to the optic nerve. The average of three single measurements was recorded for analysis. Data were analyzed using “SDLF version 4.0”, which has shown to be a reliable tool for the measurements of retinal arteriolar in vivo in humans [10].

For measurement of flicker-light-induced vasodilatation RCF was determined at baseline (after 30 minutes of rest) and after flicker light stimulation (10 Hz; Photo Stimulator 750, Siemens-Elema, AB, Germany). The repeated flashes increase retinal blood flow at least in part via NO-dependent vasodilatation and it represents a non-pharmacological tool to investigate vasodilatory capacity of retinal capillaries [23] thereby also being indicative of early vascular remodelling.

Measurement of basal NO activity of the retinal vasculature was conducted after a resting phase of 10 minutes to ensure that blood flow was at its baseline. The NO synthase inhibitor L-NMMA (Clinalfa, Läufelingen, Switzerland) was administered intravenously as a bolus infusion at a dose of 3 mg/kg of body weight over 5 minutes. Changes of RCF reflect basal NO activity of retinal vasculature, which is an independent determinant of arteriolar remodeling in the retina [24].

Measurement of vessel and lumen diameter of retinal arteriols, wall thickness and WLR were assessed using an arteriole with a size between 80 and 140 μm of the superficial retinal layer in a retinal sample of 2.56 × 0.64 × 0.30 mm, which was scanned within 2 seconds at a resolution of 256 points × 64 lines × 128 lines as described previously [7]. Analyses of diameters were performed offline with automatic fullfield perfusion imaging analysis (SLDF version 4.0 by Welzenbach) [10]. Outer arteriole diameter (AD) was measured in reflection images, and lumen diameter (LD) was measured in perfusion images. WLR as a marker of early vascular remodeling was calculated using the formula (AD-LD)/LD.

### Pulse wave analysis

The central aortic pressure waveform can be used to determine central systolic and diastolic blood pressure (BP), central pulse pressure (PP) and augmentation pressure (AP). Central PP and augmentation index (cAIx) (AP as a proportion of PP) are markers of arterial stiffness and have been shown to correlate with cardiovascular morbidity and mortality [25,26]. The central arterial waveform was derived by using the SphygmoCor™ System (AtCor Medical, Sydney, Australia). The radial artery waveform was recorded from the radial artery at the wrist, using high-fidelity applanation tonometry (Millar Instruments, Houston, Texas). The SphygmoCor™ System automatically generates the corresponding central (aortic) waveforms from an averaged radial artery waveform. From the central waveform information on central systolic and diastolic BP as well as AP and cAlx were derived. The cAlx was normalized to a heart rate of 75 beats per minute (cAlx@75).

### Pulse wave velocity

PWV is a direct measure of arterial stiffness of large arteries. For the determination of aortic PWV, waveforms of the common carotid artery and the femoral artery were obtained again using the SphygmoCor™. PWV was calculated as the distance between the suprasternal notch and the femoral artery recording site, and divided by the time interval between the feet of the flow waves.

### Statistical analysis

To perform a formal sample size calculation the primary endpoint was set to be the effect of saxagliptin compared to placebo on the change of RCF after i.v. L-NMMA application. We estimated that at least 38 fully evaluable patients would be needed (α = 0.05; β = 0.80; SD = 9%, effect size 6%). Assuming a drop-out rate of 15% and about 10% non-evaluable patients we determined a sample size of 50 subjects to be included.

Data were entered in duplicate into a Microsoft Access (Seattle, Washington) database and the analysis was performed using SPSS (release 19.0 SPSS Inc. Chicago, Illinois, USA).

Normal distribution was confirmed by Kolmogorov-Smirnow tests prior to further analyses. Normally distributed data were compared by paired t-tests and expressed as mean ± standard error of the mean (SEM). Non-parametric data (UACR) where compared using the Wilcoxon test and are presented as median and interquartile range. A two-sided P-value < 0.05 was considered statistically significant.

## Results

A total of 50 patients were recruited for the study. Four patients dropped out prior to randomization. A further four patients were excluded from the per protocol analysis because either after randomization SLDF measurements were not evaluable (n = 3) or due to high blood glucose levels that required study discontinuation for safety reasons (n = 1). Therefore the analysis was based on a total of 42 patients which had a mean age of 60.3 ± 7.2 years and 13 of these were female (31%). The average body mass index (BMI) was 30.6 ± 5.6 kg/m^2^, and the mean duration of diabetes 4 years. HbA1c prior to randomization was 6.99% (53 mmol/mol) and BP 132/79 mmHg.

By analysing our data stratified according first treatment (placebo versus saxagliptin) at baseline (week 0) as well as stratified according chronological phases (phase 1 versus phase 2) we were able to ensure successful randomization and to rule out a carry-over effect on the presented results, respectively (data not shown).

### Clinical characteristics

After (already) 6 weeks of saxagliptin treatment HbA1c was significantly lower in the saxagliptin group than in the placebo group (6.84 ± 0.15 (51 ± 1.6) versus 7.10 ± 0.17% (54 ± 1.9 mmol/mol); p < 0.001). A significant reduction with saxagliptin was also noted for postprandial glucose (9.27 ± 0.4 versus 10.1 ± 0.4 mmol/L; p = 0.001) compared to placebo. The nominal comparison in fasting blood glucose (7.21 ± 0.3 versus 7.49 ± 0.3 mmol/L; p = 0.097) did however not reach statistical significance. Adiponectin concentrations tended to be higher in the saxagliptin group (4.77 ± 0.59 versus 4.58 ± 0.54 μg/ml; p = 0.110). Total as well as LDL- and HDL-cholesterol was lower in the saxagliptin group than in the placebo group. There was no significant effect of saxagliptin on insulin levels, the HOMA index, office systolic and diastolic BP, weight, or BMI (Table 1).

**Table 1:** Clinical Characteristics (n = 42)

	**Placebo**	**Saxagliptin**	**p-value**
HbA1c (%)	7.10 ± 0.17	6.84 ± 0.15	< 0.001
Glucose postprandial (mmol/L)	10.1 ± 0.4	9.27 ± 0.4	0.001
Glucose fasting (mmol/L)	7.49 ± 0.3	7.21 ± 0.3	0.097
Adiponectin (μg/ml)	4.58 ± 0.54	4.77 ± 0.59	0.110
Insulin (pmol/L)	88.2 ± 9.7	88.4 ± 9.7	0.975
HOMA-Index	4.21 ± 0.47	4.13 ± 0.49	0.827
Office blood pressure			
Systolic (mmHg)	132 ± 2.5	131 ± 2.0	0.437
Diastolic (mmHg)	80 ± 1.3	79 ± 1.3	0.269
Weight (kg)	92.1 ± 2.6	92.4 ± 2.7	0.207
Body Mass Index (kg/m^2^)	30.6 ± 0.8	30.7 ± 0.8	0.233
Lipids			
Total cholesterol (mmol/L)	5.41 ± 0.1	5.21 ± 0.1	0.023
LDL cholesterol (mmol/L)	3.73 ± 0.1	3.55 ± 0.1	0.015
HDL cholesterol (mmol/L)	1.24 ± 0.04	1.17 ± 0.04	< 0.001
Triglycerides (mmol/L)	1.89 ± 0.1	1.84 ± 0.1	0.602

HOMA-Index, Homeostasis Model Assessment.

### Retinal circulation and arteriolar structure (Microcirculation)

**Figure 2:**
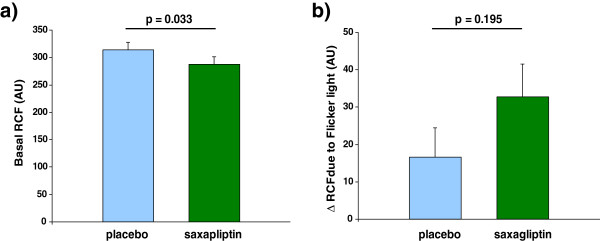
**Retinal capillary flow (RCF) after 6 weeks of treatment with placebo and saxagliptin. a**: Baseline RCF. **b**: Change of RCF due to Flicker light (vasodilatory capacity).

RCF at baseline was significantly lower after 6 weeks of treatment with saxagliptin than with placebo (288 ± 13.2 versus 314 ± 14.1 AU, p = 0.033) (Figure 2a). After flicker light stimulation no significant difference was seen in RCF between saxagliptin and placebo (323 ± 16.8 versus 331 ± 13.6 AU). Although not significant, the vasodilatory capacity (i.e. the increase of RCF) was numerical nearly two fold greater after 6 weeks of saxagliptin than after placebo (32.8 ± 8.7 versus 16.6 ± 7.9 AU; p = 0.195) (Figure 2b).

No significant difference were observed in RCF in response to infusion of L-NMMA after 6 weeks of saxagliptin compared to placebo (297 ± 13.1 versus 318 ± 12.8 AU; p = 0.116). The basal NO activity in the retinal circulation in the saxagliptin group was not significantly higher than in the placebo group (p = 0.442).

After treatment of saxagliptin for 6 weeks no significant changes were seen in WLR, wall thickness (WT) and in vessel and lumen diameter (Table 2).

**Table 2:** Parameters of micro- and macrocirculation (n = 42)

	**Placebo**	**Saxagliptin**	**p-value**
**Retinal capillary flow**			
RCF basal (AU)	314 ± 14.1	288 ± 13.2	0.033
RCF flicker (AU)	331 ± 13.6	323 ± 16.8	0.462
∆ (AU)	16.6 ± 7.9	32.8 ± 8.7	0.195
∆ (%)	6.6 ± 2.2	11.4 ± 2.5	0.176
RCF pre L-NMMA (AU)	318 ± 11.3	306 ± 13.5	0.245
RCF post L-NMMA (AU)	318 ± 12.8	297 ± 13.1	0.116
∆ (AU)	0.0 ± 6.9	- 9.0 ± 7.5	0.442
∆ (%)	0.0 ± 2.1	- 2.1 ± 2.2	0.553
**Retinal arteriolar structure**			
WLR (-)	0.38 ± 0.01	0.39 ± 0.1	0.727
WT (μm)	14.8 ± 0.5	14.8 ± 0.6	0.987
Vessel diameter (μm)	107.7 ± 1.7	106.7 ± 2.0	0.394
Lumen diameter (μm)	78.1 ± 1.2	77.0 ± 1.4	0.256
**Macrocirculation**			
Central SBP (mmHg)	124 ± 2.3	119 ± 2.3	0.038
Central PP (mmHg)	45.3 ± 2.0	41.9 ± 2.0	0.058
Central AP (mmHg)	12.4 ± 0.9	11.0 ± 1.0	0.094
cAIx@75 (%)	23.0 ± 1.2	21.8 ± 1.3	0.212
PWV (m/s)	8.86 ± 0.26	8.56 ± 0.26	0.260
UACR (mg/g creatinine)	5.0 (4.0 – 12.5)	6.0 (4.0 – 9.0)	0.285

Legend: RCF, retinal capillary flow; AU, arbitrary unit; L-NMMA, NG-monomethyl-L-arginine; WLR, wall to lumen ratio; WT, wall thickness; SBP, systolic blood pressure; PP, pulse pressure; AP, augmentation pressure; cAlx@75, central augmentation index normalized to a heart rate of 75 beats/min; PWV, pulse wave velocity; UACR, urinary albumin-creatinine ratio.

### Macrovascular circulation

**Figure 3:**
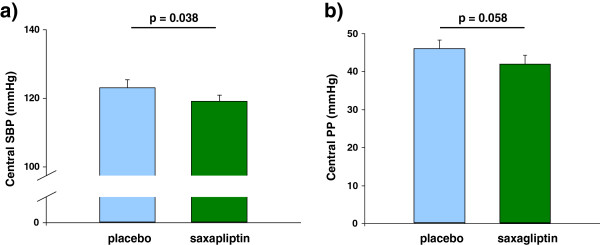
**Central hemodynamics after 6 weeks of treatment with placebo and saxagliptin. a**: Central systolic BP. **b**: Central pulse pressure.

No significant changes were seen for PWV, cAIx@75 and UACR (Table 2). Central systolic BP was significantly reduced after 6 weeks of saxagliptin (119 ± 2.3 versus 124 ± 2.3 mmHg; p = 0.038) (Figure 3a), and in accordance central PP (41.9 ± 2.0 versus 45.3 ± 2.0 mmHg; p = 0.058) (Figure 3b) and central AP (11.0 ± 1.0 versus 12.4 ± 0.9 mmHg; 0.094) tended to be lower after treatment with saxagliptin compared to placebo.

### Pre-specified subgroup analysis

To evaluate the pharmacologic action of saxagliptin more precisely, we analyzed the results of those 32 patients who responded with a reduction in postprandial blood glucose upon treatment with saxagliptin (Table 3). The effects of 6 weeks of saxagliptin on HbA1c (6.89 ± 0.2 (52 ± 2.2 mmol/l) versus 7.19 ± 0.2% (55 ± 2.2 mmol/l); p < 0.001) and postprandial glucose (9.05 ± 0.4 versus 10.4 ± 0.4 mmol/L; p < 0.001) were significant compared to placebo. RCF at baseline as well as prior to L-NMMA-infusion also was significantly decreased (RCF basal 280 ± 12.1 versus 314 ± 16.6 AU; p = 0.011; RCF pre L-NMMA 296 ± 12.3 versus 319 ± 12.4 AU; p = 0.041). Similar to the whole study population the vasodilatory capacity upon flicker light was numerically two-fold greater compared to placebo (35.6 ± 10.9 versus 19.1 ± 10.1 AU, p = 0.306). Compared to placebo saxagliptin had no significant effects on RCF in response to the infusion of L-NMMA and no influence on retinal arteriolar structural parameters in this subgroup (Table 3).

**Table 3:** Subgroup analysis with patients showing a decrease in postprandial blood glucose (n = 32)

	**Placebo**	**Saxagliptin**	**p-value**
**Clinical characteristics**			
HbA1c (%)	7.19 ± 0.2	6.89 ± 0.2	< 0.001
Glucose postprandial (mmol/L)	10.4 ± 0.4	9.05 ± 0.4	< 0.001
Glucose fasting (mmol/L)	7.60 ± 0.4	7.21 ± 0.3	0.077
Office SBP (mmHg)	132 ± 2.8	131 ± 2.6	0.564
Office DBP (mmHg)	80 ± 1.4	79 ± 1.4	0.577
Weight (kg)	90.6 ± 2.4	91.0 ± 2.5	0.162
Total cholesterol (mmol/L)	5.34 ± 0.1	5.08 ± 0.1	0.011
LDL cholesterol (mmol/L)	3.73 ± 0.1	3.50 ± 0.1	0.013
HDL cholesterol (mmol/L)	1.19 ± 0.03	1.14 ± 0.03	< 0.001
Triglycerides (mmol/L)	1.85 ± 0.2	1.83 ± 0.2	0.802
**Retinal capillary flow**			
RCF basal (AU)	314 ± 16.6	280 ± 12.1	0.011
RCF flicker (AU)	334 ± 16.3	319 ± 18.7	0.243
∆ (AU)	19.1 ± 10.1	35.6 ± 10.9	0.306
∆ (%)	7.3 ± 2.7	12.1 ± 3.1	0.299
RCF pre L-NMMA (AU)	319 ± 12.4	296 ± 12.3	0.041
RCF post L-NMMA (AU)	309 ± 12.6	297 ± 14.2	0.371
∆ (AU)	- 9.8 ± 6.5	1.1 ± 8.2	0.344
∆ (%)	- 2.8 ± 2.1	0.7 ± 2.6	0.339
**Retinal arteriolar structure**			
WLR (-)	0.38 ± 0.01	0.38 ± 0.02	0.837
WT (μm)	14.8 ± 0.6	14.5 ± 0.7	0.564
Vessel diameter (μm)	108 ± 2.1	106 ± 2.5	0.261
Lumen diameter (μm)	78 ± 1.5	77.4 ± 1.8	0.362
**Macrocirculation**			
Central SBP (mmHg)	123 ± 2.5	119 ± 2.0	0.080
Central PP (mmHg)	46.0 ± 2.3	41.9 ± 2.4	0.051
Central AP (mmHg)	13.3 ± 1.1	11.7 ± 1.1	0.122
cAlx@75 (%)	24.7 ± 1.3	23.6 ± 1.4	0.367
PWV (m/s)	8.39 ± 0.24	8.38 ± 0.27	0.971
UACR (mg/ g creatinine)	5.0 (4.0 – 11.0)	6.0 (3.25 – 9.0)	0.740

Legend: SBP, systolic blood pressure; DBP, diastolic blood pressure; RCF, retinal capillary flow; AU, arbitrary unit; L-NMMA, NG-monomethyl-L-arginine; WLR, wall to lumen ratio; WT, wall thickness; PP, pulse pressure; AP, augmentation pressure; cAlx@75, central augmentation index normalized to a heart rate of 75 beats/min; PWV, pulse wave velocity; UACR, urinary albumin-creatinine ratio.

## Discussion

There is accumulating evidence that the first stage of early diabetic retinopathy is characterized by increased retinal blood flow. Kohner et al. described already in 1975 an increase in retinal blood flow in diabetics without or with mild retinopathy [27] and Grunwald et al. observed that high blood glucose is associated with a decrease in retinal vascular response [28]. Thus, high blood glucose interferes with autoregulation of the retinal vessels and causes a constantly increased blood flow. This may result in damages of the endothelial lining of blood vessels which is a key factor in the development of diabetic retinopathy [3]. These microcirculatory changes in the retina resembles those repeated observed in the microcirculation of the kidneys [29,30].

Our major finding was that RCF at baseline was significantly lower after 6 weeks of saxagliptin treatment than in type-2 diabetic patients receiving placebo, whereas WLR was unchanged. Results were pronounced in a subgroup of patients with a reduction of postprandial glucose taken as a marker of the pharmacological action of saxagliptin. By applying this prespecified subgroup analysis, we thought to eliminate non-compliance (pill counting was in all patients > 80%) and unresponsiveness of type-2 diabetic patients to the pharmacological effects of DPP-4 inhibitors. Our data indicate that treatment with saxagliptin resulted in a lower RCF which should be considered as a sign towards normalization of RCF in early type-2 diabetes. Our results are in disagreement with a previous open label trial that reported a mean increase in RCF from baseline to 24-weeks with vildagliptin compared to glimepiride on top of metformin [31]. There are, however, a number of significant differences in study design and population that prohibit a valid comparision with our study: patients had long-standing diabetes, higher baseline HbA1c values, higher BMI and received combination therapy. The study was open label, not double blind (like our trial) and the absolute values of RCF were only 1/3 of RCF values reported by other groups [11,32], including their own previous work [33] despite using the same methodology, thereby questioning the correctness of the reported data.

Between treatment groups we found no significant difference in the change of RCF in response to L-NMMA treatment. While L-NMMA inhibits basal NO synthase activity flicker light stimulation results in partially NO dependent vasodilation and overall serves as a vasodilatory test of retinal arterioles. Dorner et al found that about 50% of the flicker light-induced increase in retinal arteriolar and venular vasodilatation can be blocked by L-NMMA infusion [34]. The retinal microvascular response to flicker light has been described to be impaired under certain pathological conditions such diabetes [35,36] or hypertension [6,37,38]. It was suggested that in patients with diabetes and/or hypertension, endothelial dysfunction and the restricted capability of the endothelial cell to secrete NO might cause a disturbed microvascular blood flow. Given that our patients were diagnosed with type-2 diabetes mellitus (defined by fasting glucose ≥ 7.0 mmol/L or HbA1c ≥ 6.5% (48 mmol/mol) or receiving anti-diabetic pharmacotherapy), this might have impacted the ability of the retinal microvasculature to respond to these stimuli.

We hypothesized that the ability of the retinal microvascular respond to these stimuli can be improved by saxagliptin [18-22]. The vasodilatory capacity was two-fold increased in patients with flicker light exposure receiving saxagliptin, but this two-fold increase did not reach statistical significance due to the high variation of the vasodilatory response. In our previous work we had to include 139 patients to demonstrate a significant difference of the vasodilatory capacity between normotensive and hypertensive subjects [38], a finding that had been repeated shown in other vascular beds. Our finding of a non significant two-fold increase of vasodilation suggest that, if any, vasodilatory capacity of flicker light (a parameter of early vascular remodeling of the retinal arterioles) may improve after DPP-4 inhibition with saxagliptin. Previous findings in an animal model showed that vildagliptin inhibited inflammatory and thrombogenic reactions in the retina of Otsuka Long-Evans Tokushima Fatty (OLEFT) rats supports the beneficial effects of DDP-4 inhibition on diabetic retinopathy [39].

This beneficial effect of saxagliptin in the retina was observed in parallel to other vascular signals indicative of improvement, i.e. normalization of vascular function. In the macrocirculation central (aortic) systolic pressure decreased significantly (but not office BP measured at brachial level), and central PP and AP tended to decrease towards normal values. These discrete changes in the macrocirculation points towards to a normalization of wave reflection in the arterial tree in the saxagliptin group. While one should be cautious in extrapolating these results to potential macrovascular benefits of DPP-4 inhibitors overall, or saxagliptin in particular, there is a plausible mechanistic link between these observations and data recently published by Rathmann [40] and Monami [41] demonstrating a macrovascular benefit of these drugs. In a pooled analysis of phase III clinical trials the DDP-4 inhibitor linagliptin achieved an improved glycemic control and was well tolerated in a population at high risk for micro- and macrovascular complications [42].

Recently The Saxagliptin Assessment of Vascular Outcomes Recorded in Patients with Diabetes Mellitus (SAVOR) - Thrombolysis in Myocardial Infarction (TIMI) 53 trial comprising 16,492 patients with type 2 diabetes who had a history of, or were at risk for cardiovascular events, a reported unchanged risk of the pre-specified macrovascular (e.g. cardiovascular) composite primary endpoint, but an increased rate of hospitalization for heart failure, which is not explainable and subject of ongoing analysis. The former is not surprising, since with a median of 2.1 years of follow-up no such effect can be expected in this short period. Interestingly, saxagliptin treatment resulted in both less worsening and higher rate of normalization of microalbuminuria (both p < 0.001), indicating an improvement of microvascular damage [43].

This is in line with previous findings. In the Steno-2 study, a multifactorial approach of intensive treatment significantly reduced microvascular complications (including diabetic nephropathy and retinopathy) already after a mean monitoring period of 3.8 years [44], whereas the number of macrovascular events was significantly reduced after 13.3 years [45]. Moreover, The Action in Diabetes and Vascular Disease: Preterax and Diamicron Modified Release Controlled Evaluation (ADVANCE) trial with a median follow–up of 5 years showed that intensive control reduced major microvascular events, primarily because of a reduction in the incidence of nephropathy, whereas major macrovascular events were not significantly effected [46].

Our study was not designed for determining the underlying mechanism, but looking at the literature and our own data it appears that various DPP-4 inhibitors are also able to improve endothelial function pointing to a class effect. Previously, it was shown that alogliptin increased both postprandial endothelial function and lipidemia, indicating anti-atherogenic effects [47]. However, animal experiments and human studies have shown that downstream effects of DDP-4 inhibition, namely GLP-1, impacts on vasculature via GLP-1 receptor in-, and dependent pathways [18-21]. DPP-4 cleaves not exclusively GLP-1, but also other known vascular effective peptides like stromal cell-derived factor 1α (SDF-1α). Furthermore, it was shown that sitagliptin imcreases endothelial progenitor cells (EPCs) in patients with type 2 diabetes, indicating an improvement of endothelial function [48].

## Conclusions

To sum up, treatment with saxagliptin for 6 weeks resulted in a reduction of RCF in microcirculation and reduced central systolic pressure. In accordance with these data, we noted signals that the vasodilatory capacity of the retinal arterioles may increase, and central PP and AP decrease. Thus, data suggest that compared to placebo the DPP-4 inhibitor saxagliptin may reverse early hemodynamic and vascular remodeling processes in type-2 diabetes.

## Abbreviations

AD: Outer arteriole diameter; ADVANCE: The action in diabetes and vascular disease: Preterax and diamicron modified release controlled evaluation; cAIx(@75): Central augmentation index (normalized to a heart rate of 75 beats per minute); AP: Augmentation pressure; AU: Arbitrary units; BP: Blood pressure; DDP-4: Dipeptidyl peptidase-4; ESENDI: Effects of saxagliptin on ENdothelial function in patients with type-2 diabetes; GLP-1: Glucagon-like-peptide 1; HbA1c: Glycated hemoglobin; HOMA: Homeostasis model assessment; LD: Lumen diameter; L-NMMA: NG-monomethyl-L-arginine; NO: Nitric oxide; RCF: Retinal capillary flow; PP: Pulse pressure; PWV: Pulse wave velocity; SAVOR-TIMI: The saxagliptin assessment of vascular outcomes recorded in patients with diabetes mellitus - thrombolysis in myocardial Infarction 53 trial; SLDF: Scanning laser Doppler flowmetry; UACR: Urinary albumin-to-creatinine ratio; WLR: Wall-to-lumen ratio; WT: Wall thickness.

## Competing interests

Saxagliptin is a drug developed by Bristol-Myers Squibb and Astra Zeneca. PB and RES receive research funding and consulting honoraria from both companies beyond the scope of the present study. All other authors have no competing interests to disclose.

## Authors’ contributions

CO participated in conception of research design, researched data, analyzed data, wrote the manuscript and reviewed/edited the manuscript. UR participated in conception of research design, researched data, contributed to the discussion and reviewed/edited the manuscript. SS researched data and reviewed/edited the manuscript. IK researched data and reviewed/edited the manuscript. SF researched data and reviewed/edited the manuscript. PB participated in data analysis and wrote the manuscript. JMH researched data, contributed to the discussion and reviewed/edited the manuscript. RES participated in conception of research design, analyzed data, wrote manuscript and reviewed/edited the manuscript. All authors read and approved the final manuscript.
